# Supplementary material for: Altered microRNA Transcriptome in Cultured Human Liver Cells upon Infection with Ebola Virus
Source: Int J Mol Sci. 2021 Apr 6;22(7):3792. doi: 10.3390/ijms22073792 (PMC8038836; doi:10.3390/ijms22073792)
Supplement: Supplementary file 1 [file ijms-22-03792-s001.zip › Supplementary_File/C_ GO_Analysis_Results/16-30nt_go_Reston-24h-Huh7_vs_Control-24h-Huh7_down.mature_mirna_targets/GeneOntology_FoldEnrichment.pdf]

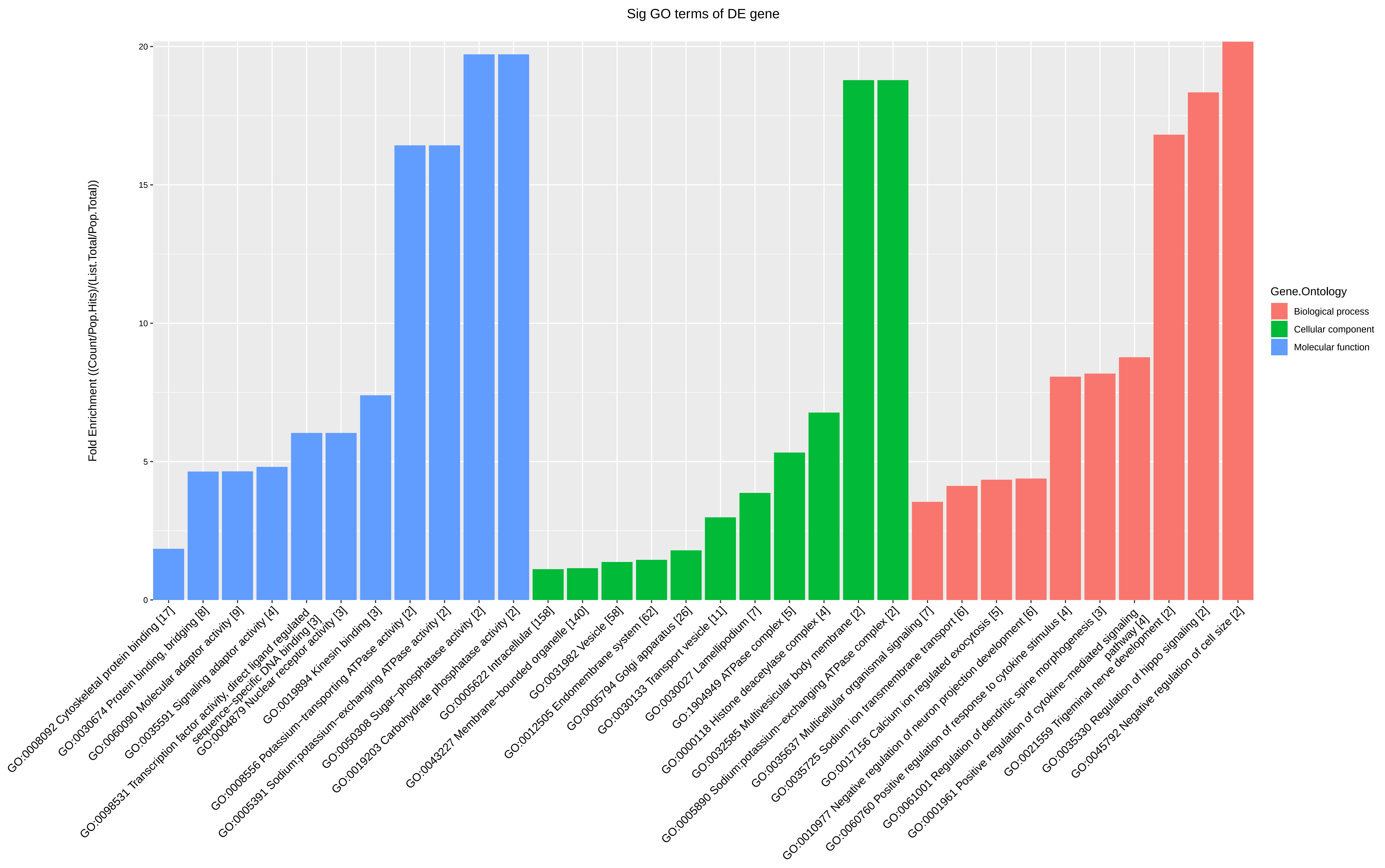

Sig GO terms of DE gene
Fold Enrichment ((Count/Pop.Hits)/(List.Total/Pop.Total))
0
5
10
15
20
Gene.Ontology
Biological process
Cellular component
Molecular function
GO:0008092 Cytoskeletal protein binding [17]
GO:0030674 Protein binding, bridging [8]
GO:0060090 Molecular adaptor activity [9]
GO:0035591 Signaling adaptor activity [4]
GO:0098531 Transcription factor activity, direct ligand regulated sequence−specific DNA binding [3]
GO:0004879 Nuclear receptor activity [3]
GO:0019894 Kinesin binding [3]
GO:0008556 Potassium−transporting ATPase activity [2]
GO:0005391 Sodium:potassium−exchanging ATPase activity [2]
GO:0050308 Sugar−phosphatase activity [2]
GO:0019203 Carbohydrate phosphatase activity [2]
GO:0005622 Intracellular [158]
GO:0043227 Membrane−bounded organelle [140]
GO:0031982 Vesicle [58]
GO:0012505 Endomembrane system [62]
GO:0005794 Golgi apparatus [26]
GO:0030133 Transport vesicle [11]
GO:0030027 Lamellipodium [7]
GO:1904949 ATPase complex [5]
GO:0000118 Histone deacetylase complex [4]
GO:0032585 Multivesicular body membrane [2]
GO:0005890 Sodium:potassium−exchanging ATPase complex [2]
GO:0035637 Multicellular organismal signaling [7]
GO:0035725 Sodium ion transmembrane transport [6]
GO:0017156 Calcium ion regulated exocytosis [5]
GO:0010977 Negative regulation of neuron projection development [6]
GO:0060760 Positive regulation of response to cytokine stimulus [4]
GO:0061001 Regulation of dendritic spine morphogenesis [3]
GO:0001961 Positive regulation of cytokine−mediated signaling pathway [4]
GO:0021559 Trigeminal nerve development [2]
GO:0035330 Regulation of hippo signaling [2]
GO:0045792 Negative regulation of cell size [2]
